# Correction: The impact of housing prices and land financing on economic growth: Evidence from Chinese 277 cities at the prefecture level and above

**DOI:** 10.1371/journal.pone.0304494

**Published:** 2024-05-23

**Authors:** Qian Sun, Sohail Ahmad Javeed, Yong Tang, Yan Feng

The published funding statement is inaccurate. The correct funding statement is as follows: This work was supported by grants from the National Natural Science Foundation of China General Program (72373037), Social Science Foundation of Hunan Province Key Program (22ZDB080), Provincial Natural Science Foundation of Hunan (2022JJ30116).

## References

[1] Sun Q, Javeed SA, Tang Y, Feng Y (2024) The impact of housing prices and land financing on economic growth: Evidence from Chinese 277 cities at the prefecture level and above. PLoS ONE 19(4): e0302631. 10.1371/journal.pone.0302631. 38687821 PMC11060597

